# Recovering From COVID-19: A Transatlantic Comparison of Fiscal Policy

**DOI:** 10.1007/s10272-021-0989-2

**Published:** 2021-08-05

**Authors:** Daniel Gros

**Affiliations:** Centre for European Policy Studies (CEPS), 1 Place du Congrès, 1000 Brussels, Belgium

## Abstract

A chasm has opened up across the Atlantic in terms of fiscal policy.

A chasm has opened up across the Atlantic in terms of fiscal policy. In the US, the fiscal deficit was about 16% of GDP in 2020, and it is projected to stay around this value in 2021. By contrast, the deficits in the euro area are only half this amount (7% and 8% of GDP) in both these years.

How should one understand this stark difference? An answer to this question has to take into account the extraordinary circumstances of the COVID-19-induced recession. This recession was different because it did not result from overinvestment in a specific sector (e.g. housing) coupled with overstretched borrowers and stressed financial institutions.

The financial system is at present not a source of concern. After an initial period of extreme volatility, financial markets recovered with stock market prices at historically high levels and risk premia generally at historically low levels. There is thus no problem with the availability of credit to solvent borrowers.

However, during the lockdown period, many workers could not do their jobs as numerous enterprises had to close and services that required close physical contact were either closed by government or consumers were simply too afraid to venture out. This meant that households (and enterprises) lost a large part of their income. Governments had to step in to keep them afloat. After all, the government is the "insurer of last resort". The need for fiscal support from the insurance function can be estimated roughly by the size of the output gap that opened up in 2020/21. The loss of GDP in these two years is equal to the value added which was not produced because of COVID-19 restrictions.

### Deficit versus the output gap: Replacement income versus demand stimulus

One can thus divide the observed deficits during the two coronavirus pandemic years into two parts: one part, equal to the output gap, provided replacement income. The remainder could be viewed as standard deficit spending with the aim of fostering demand.

Figure 1 shows the data for both the output gap and the (general government) deficit for the three years 2020-2022 as a percentage of (potential) GDP. It is apparent that the deficits for the US are much larger than could be justified by the need to provide replacement income. In the euro area, the deficits are lower, and the output gap is larger (especially in 2020), with the result that a much larger part of the observed (and planned) deficits consist of providing replacement income.

The large part of deficits not needed to provide replacement income is illustrated in Figure 2. It shows the size of the "excess deficit", i.e. the difference between the actual deficit and the output gap (again as a percentage of potential GDP). Panel A allows for a more direct comparison of the aggregate demand effort than the picture of the two components separately in Figure 1.

Looking directly at the difference between deficits and the output gap, the difference between the US and the euro area is even starker. While only a very small part of the US deficit of 16% of GDP for 2020 can be explained by the need to provide a replacement income, a much larger part of the smaller euro area deficit is needed for this purpose. In 2021, the US demand impulse close to 17% of GDP is almost four times larger than the 4.7% in the euro area. A significant difference is also projected to remain in 2022, when the aggregate demand aspect of fiscal policy will amount to 8.2% of GDP in the US, against 3.5% of GDP in the euro area.

Panel B shows that there are large differences between individual euro area countries, during all three years, however, no euro area country shows values close to those of the US. For example, Spain seems to have concentrated its fiscal effort in 2020, whereas most of the others have a peak in 2021. Germany plans apparently to return to its prudent fiscal policy stance already in 2022.

Figure 1
**Deficit and output gap**

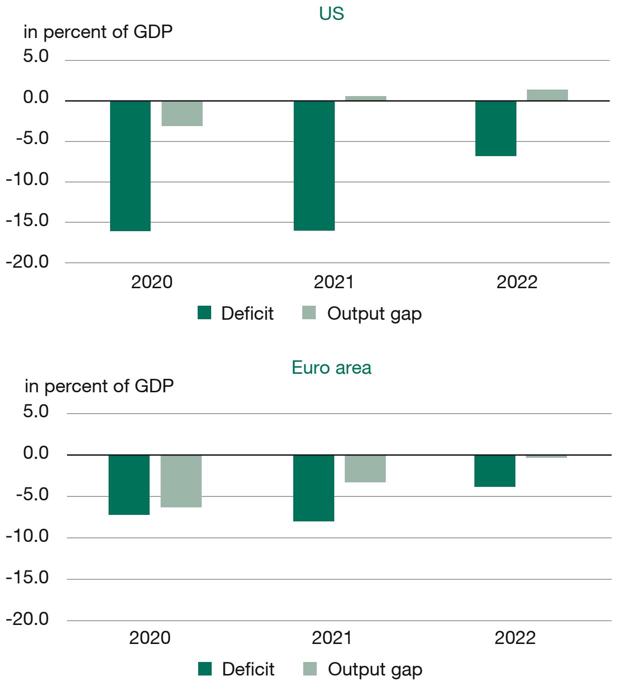

Sources: IMF and European Commission (output gap), AMECO database (deficits), June 2021.

Figure 2
**Deficit in excess of output gap**

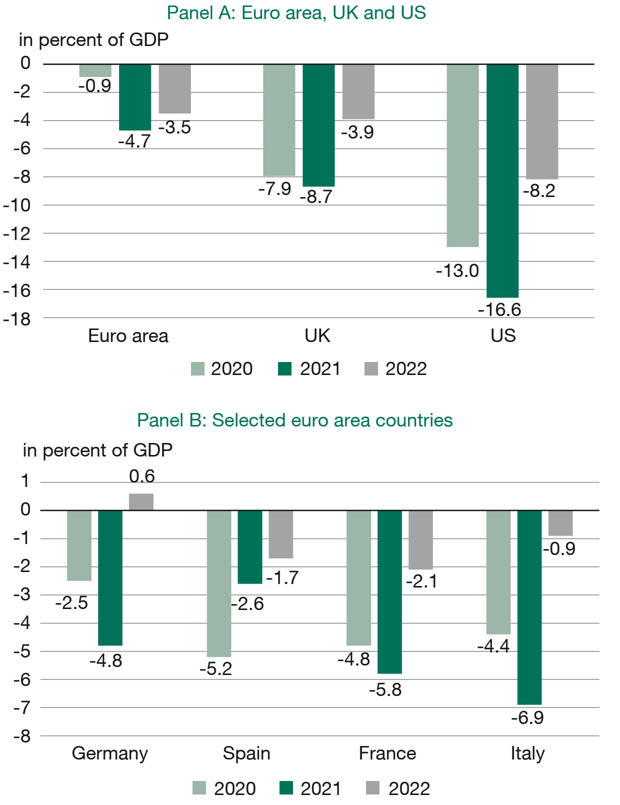

Sources: IMF and European Commission (output gap), AMECO database (deficits), June 2021.

## Will demand stimulus work?

The modern view of fiscal policy is that it works by providing cash or liquidity to “hand-to-mouth” consumers, i.e. households that are constrained in their spending by their current disposable income. Consumers who do not face a liquidity constraint are unlikely to consume additional transfers provided by the government because they have to think about the future taxes with which the government will have to finance these transfers. But the models typically assume that a fraction of households are constrained by the availability of liquidity and that these households respond to higher transfers by consuming more. This feature is embedded into most of the models economists use to evaluate policy (see, for example, Burgert et al., 2020).

This type of reasoning implies that in a recession when unemployment is high, fiscal policy can foster an increase in demand by providing households with an income to spend. Households are likely to spend a large proportion of their current income on consumption when they can expect that future income will be higher. Expecting future incomes to be higher than current ones is natural for the unemployed in a recession. They can expect to earn more as the economy recovers. This simple approach also explains why multipliers (i.e. the impact of fiscal policy on output) are higher during recessions. When unemployment is low, there are fewer households around that require support to increase their spending today because their present income is already high relative to expected future incomes.

However, the COVID-19 recession is special, and the reactions of governments were very strong, as illustrated above. With deficits much larger than the output gap, household disposable income actually increased in many cases. However, consumption spending could not increase because of the COVID-19 restrictions. As a result, household savings increased. This can be seen in the large accumulation of financial assets already in 2020, illustrated in Figure 3. The financial assets of households in the euro area increased by almost 50%, from about €2,000 per capita to over €3,000 (left-hand panel). The right-hand panel of this chart shows that the increase in financial assets continues even after the large jump during the acute lockdown phase of early 2020.

This implies that there must be far fewer liquidity constrained consumers today than before the crisis. The standard models would thus imply that the impact of defi-

Figure 3

**Household investments in financial assets and contributions by components**

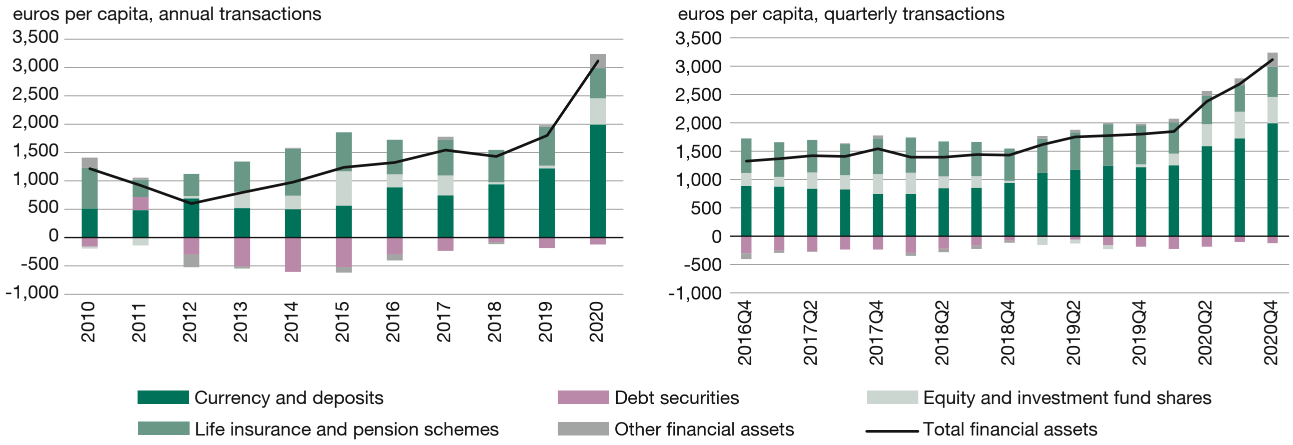

Sources: Eurostat und ECB calculations, 2021.

cit spending on demand should be much smaller than before. Apart from this technical point, it is clear that households now have the liquidity they need to spend.

A return to pre-pandemic consumption patterns is of course not certain. Households might remain cautious because of the memory of the uncertain times they just had to endure (Kozlowski et al., 2020). However, even if this were true, it would still imply that large fiscal deficits in excess of the need for replacement income should have a limited effect on the economy.

What could be the reason for the large differences in fiscal policy? Two mechanisms might be at play: confirmation bias and prisoner's dilemma.

## Confirmation bias

One key underlying reason for this drifting apart is "confirmation bias" (Rodrik, 2020; Klayman, 1995), which results from a human tendency to find an affirmation of one's long-held beliefs in a crisis.

In the US, this crisis is seen by many through the lens of the 2008/9 crisis, when, according to perceived wisdom, the fiscal response was not large enough. "Go big" is the lesson learnt from that crisis, which should be applied today as well (Krugman, 2021).

In the euro area, this crisis is seen in Italy and Spain through the lens of the debate about Eurobonds, i.e. common debt issuance. The Southern members of the euro area feel vindicated in their demand for a common debt instrument and a common fiscal stabilisation mechanism. The Germans, by contrast, feel that their prudent approach to fiscal policy was vindicated by the crisis, because years of balanced budgets mean that their government can now spend much more on helping German workers and enterprises to overcome it.

All these ways of confirming long-held beliefs about what should be done have their weaknesses. The argument that the fiscal response to the 2009 recession overlooks the fact that the COVID-19 recession is different because it is caused by an exogenous, sectoral shock, which implies that fiscal policy becomes less potent (Gros, 2020; Guerrieri et al., 2020). Moreover, as argued here, consumers have accumulated large liquid balances, making it unlikely that further transfers from the government will be spent immediately.

Whatever the strength of the arguments, confirmation bias tends to harden the opposing sides' positions, and this seems to be happening within the US political system as well.

## Prisoner's dilemma

For some political forces in the US, mainly in the current administration, the question of the effectiveness of fiscal policy in terms of fostering demand and employment today is secondary. They argue that the US needs a stronger social security system and that many of the elements in the second "stimulus" package of 2021 should be made permanent. This would require a permanent increase in (federal) expenditures, but any increase in taxes is anathema to the Republican opposition, which has been able to block any sustained increase in taxes so far. As the rules of procedure are slightly different for expenditures than for tax increases (and the incentives for individual Sena-

Figure 4
**General government expenditure and revenues**

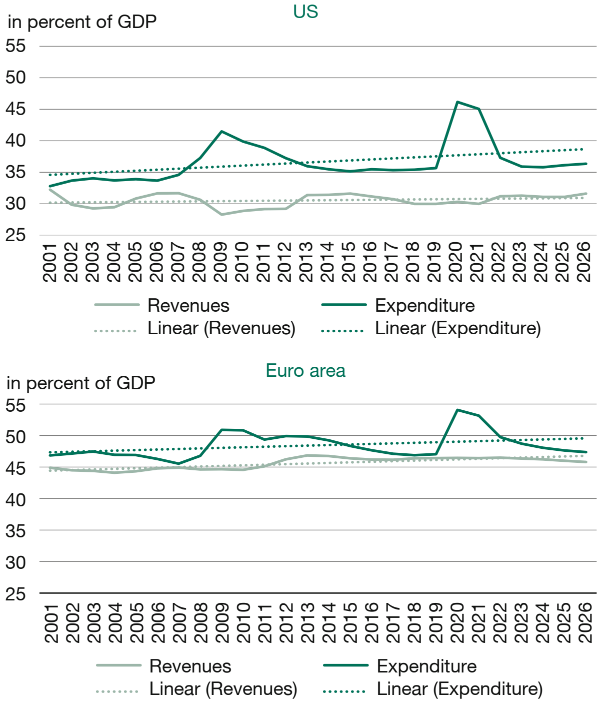

Sources: IMF, WEO database, April 2021.

tors and Representatives of Congress also differ), it has proven possible to increase expenditure, but not to increase taxes. This is unlikely to change soon, leading to a situation which has been called a "prisoner's dilemma" (Alesina and Tabellini, 1990). As mentioned above, the administration is combining the lesson learnt from the Obama administration that any fiscal stimulus has to be big and that one should use a crisis to try push through long-held plans.

Figure 4 shows that in the US, expenditures and revenues (of general government, thus taking into account not only the federal level) have been drifting apart for a long time. Since 2001, the US has not had a single year without a deficit. The reason for this is that expenditure has been consistently above the threshold for revenues that is apparently politically acceptable. Expenditure has drifted upwards from 35% of GDP to close to 40%, with an extraordinary peak of 45% in 2020/21. By contrast, revenues have not kept pace, remaining mostly within a narrow range of 30%-32% of GDP. The US is likely to continue to run large deficits as long as this political equilibrium persists.

For the euro area, by contrast, expenditure and revenues are much closer (and higher than in the US) and neither of them shows a persistent upward or downward trend.

## Conclusions

Discussion of the consequences of the US' extraordinary fiscal policy tend to focus on their short-term impact on demand and potential inflation. However, the impact of even very large transfer payments to families might be limited because households have already accumulated large liquid balances, which they could spend any time.

Moreover, this entire debate might be misleading because it neglects longer-term trends. When comparing fiscal policy in the US and the euro area in the phase of economic recovery from the coronavirus pandemic, one must take into account the very different starting points. Both areas went into the COVID-19 shock with a robust economy and low unemployment. But the US was already at that time running a deficit of over 5% of GDP (and had been doing so for a long time), whereas the euro area was close to balance. This different starting point constitutes an often-overlooked key transatlantic difference.

The fact that the US was already running large fiscal deficits before the COVID-19 shock, even at times when unemployment had fallen to historical lows, points to a more fundamental problem, namely a drifting apart of the expenditures that seem politically expedient and the revenues that can be collected. The real issue for US fiscal policy is thus not a short-term one of speeding up the recovery from the coronavirus shock, but a more fundamental long-term one of political partisanship which prevents agreement on the measures needed to stabilise public finances.

In the euro area, the situation seems different: Government expenditure and revenues are much higher than in the US, but they are not drifting apart. Here, the main issue is the more traditional and short-term one, namely how to accompany and speed up the recovery.
